# Novel bi-allelic variants of *CHMP1A* contribute to pontocerebellar hypoplasia type 8: additional clinical and genetic evidence

**DOI:** 10.3389/fneur.2023.1228218

**Published:** 2023-09-18

**Authors:** Tiantian He, Huaqin Sun, Bocheng Xu, Haibo Qu, Xiaotang Cai, Hui Zhou, Yanyan Liu, Ziyuan Lin, Xuemei Zhang

**Affiliations:** ^1^Department of Medical Genetics, Prenatal Diagnosis Center, West China Second University Hospital, Sichuan University, Chengdu, China; ^2^Department of Obstetrics and Gynecology, West China Second University Hospital, Sichuan University, Chengdu, China; ^3^Key Laboratory of Birth Defects and Related Diseases of Women and Children (Sichuan University), Ministry of Education, Chengdu, China; ^4^SCU-CUHK Joint Laboratory for Reproductive Medicine, West China Second University Hospital, Sichuan University, Chengdu, China; ^5^Department of Radiology, West China Second University Hospital, Sichuan University, Chengdu, China; ^6^Department of Rehabilitation, West China Second University Hospital, Sichuan University, Chengdu, China

**Keywords:** bi-allelic variants, *CHMP1A*, pontocerebellar hypoplasia, polyhydramnios, phenotype, genotype

## Abstract

Pontocerebellar hypoplasia type 8(PCH8) is a rare neurodegenerative disorder, reportedly caused by pathogenic variants of the *CHMP1A* in autosomal recessive inheritance, and *CHMP1A* variants have also been implicated in other diseases, and yet none of the prenatal fetal features were reported in PCH8. In this study, we investigated the phenotype and genotype in a human subject with global developmental delay, including clinical data from the prenatal stage through early childhood. Prenatally, the mother had polyhydramnios, and the bilateral ventricles of the fetus were slightly widened. Postnatally, the infant was observed to have severely delayed psychomotor development and was incapable of visual tracking before 2 years old and could not fix on small objects. The young child had hypotonia, increased knee tendon reflex, as well as skeletal malformations, and dental crowding; she also had severe and recurrent pulmonary infections. Magnetic resonance imaging of the brain revealed a severe reduction of the cerebellum (vermis and hemispheres) and a thin corpus callosum. Through whole exome sequencing and whole genomics sequencing, we identified two novel compound heterozygous variations in *CHMP1A* [c.53 T > C(p.Leu18Pro)(NM_002768.5) and exon 1 deletion region (NC_000016.10:g.89656392_89674382del)]. cDNA analysis showed that the exon1 deletion region led to the impaired expression, and functional verification with zebrafish embryos using base edition indicated variant c.53 T > C (p.Leu18Pro), causing dysplasia of the cerebellum and pons. These results provide further evidence that *CHMP1A* variants in a recessive inheritance pattern contribute to the clinical characteristics of PCH8 and further expand our knowledge of the phenotype and genotype spectrum of PCH8.

## 1. Introduction

Pontocerebellar hypoplasias (PCH) is a heterogeneous group of rare autosomal recessive neurodegenerative disorders characterized by reduced volume of the pons and cerebellum (1, 2). Pontocerebellar hypoplasia type 8 (PCH8) (MIM #614961), first reported by Mochida et al. (2), is caused by pathogenic variations in the *CHMP1A* gene in autosomal recessive inheritance. The phenotype of PCH8, as evidenced by brain magnetic resonance imaging (MRI), includes pontocerebellar hypoplasia, decreased cerebral white matter, and a thin corpus callosum, which, together, result in severe psychomotor delay, abnormal movements, hypotonia, and variable visual defects, and so on (2). *CHMP1A* is located on chromosome 16q24.3, spans 13,274 bp, and contains seven exons, encoding a 196-amino acid protein (2), which has been named charged multivesicular body protein 1A/chromatin modifying protein 1A (*CHMP1A*, MIM *164010). To date, two variants of the gene, from three families, have been associated with PCH8 (2, 3), and other reports suggest that variations in *CHMP1A* may be associated with autism spectrum disorder (4–6) or are candidate genes for late-onset Parkinson’s disease (7). Thus, more research is necessary in order to understand the effects of *CHMP1A* on neurological diseases.

In the present study, we describe the clinical characteristics of a patient who was found to have compound heterozygous variants of *CHMP1A*; further verification of functional deficits confirmed the pathogenicity of these two variants. We conclude that the pathogenic variants of *CHMP1A* documented in the present study lead to PCH8 in recessive inheritance; thus, the genotype and phenotype spectrum of PCH8 is expanded with these findings.

## 2. Materials and methods

### 2.1. Clinical materials

Blood samples were obtained from the affected individual and her parents. Genomic DNA was extracted from peripheral blood according to the kit manufacturer’s instructions (CWBIO, Beijing, China), and RNA was isolated by using the RNA pure Blood Kit (CWBIO, Beijing, China). The clinical data were collected and evaluated by a multidisciplinary team of geneticists, pediatricians, and radiologists, and the individual’s phenotype was longitudinally and systematically evaluated. The studies involving human participants were reviewed and approved by the Medical Ethics Committee of West China Second University Hospital, Sichuan University. Written informed consent to participate in this study was provided by the participant’s legal guardian.

### 2.2. Genetic testing

Trio whole exome sequencing was performed on blood samples from the proband and the parents, using the NanoWES Human Exome V1 (Berry Genomics, Beijing) following the manufacturer’s protocol. Whole genome sequencing was performed on the sample obtained from proband to confirm the exon1 deletion region and the variant in *CHMP1A* suggested by whole exome sequencing. Using the library construction kit provided by MyGenostics (MyGenostics Inc., Chongqing, China). The detailed procedure of whole genome sequencing is in Supplementary Data sheet 1.

### 2.3. cDNA analysis

The expression profile of the *CHMP1A* exon1 deletion region was evaluated based on total RNAs from the proband and her parents. Total RNA was reverse transcribed with the HiFiscript cDNA Synthesis kit (CWBIO, Beijing, China) according to the manufacturer’s instructions. Quantitative polymerase chain reaction (qPCR) reactions were performed in triplicate, using gene-specific primers [5′- AAGTCCAGCAGGAAGAAAGT-3′(sense), 5′-CTGGAACTGCAACTGACTAG-3′(antisense)] for exon one, and [5′-AGCTTGGTCGGTTCGATCG-3′(sense), 5′-CATCCGAAGCCAGTTCACAC-3′(antisense)] for exon three, SYBR Green Q-PCR Master Mix (Thermo Fisher Scientific, 00850445) on a RT-PCR System (Thermo Fisher Scientific, 7500 Real-Time PCR Systems) following the manufacturer’s instructions. Results were normalized versus the expression of the β-Actin gene.

Additionally, Sanger sequencing of exon 3 at the level of cDNA and DNA was performed using PCR, amplified using 2 × TSINGKE Master Mix (Tsingke, Beijing). The detailed procedure of Sanger sequencing is in Supplementary material 1.

### 2.4. Zebrafish functional verification of variant *Leu18Pro*

#### 2.4.1. Zebrafish embryo manipulations and whole-mount *in situ* hybridization

All animals were handled in accordance with the Guide for the Care and Use of Laboratory Animals approved by the Institutional Animal Care and Use Committee of Sichuan University. Zebrafish embryo microinjection, whole-mount *in situ* hybridization, and statistical analysis were performed as previously described (8).

#### 2.4.2. Zebrafish base editing

The zebrafish *chmp1a* gene mutation Leu18Pro was edited using the tool ZSpRY-ABE8e, and the target sequence (gRNA) was designed according to the description by Liang et al. (9) gRNA with chemical modifications comprising 2′-O-methyl-3′-phosphorothioate (MS) at both ends was synthesized using GenScript.^1^ One-cell stage zebrafish embryos were injected with 2 nL of a solution containing 100 ng/μL ZSpRY-ABE8e mRNA and 50 ng/μL gRNA mixture. Three days post-injection, we examined potential mutation events by PCR. The primers for a flanking sequence to the mutant site were as follows: forward, CTGAAACTCTGGCCAGTGACG; and reverse, ATGTTTTGGTCTCTCTCTCTCTCCT. Three months post-injection, when the fish reached sexual maturity, we incised the tail fin to obtain PCR samples, each of which was evaluated with the previously mentioned primers and to obtain a founder fish (F0). The F0 founder fish was then mated with wild type to obtain an F1 heterozygous line.

#### 2.4.3. PCR template preparation for embryo and tail fin

We placed five embryos or cut tail fins in a tube and added 50 μL of lysis buffer (10 mM Tris pH 8.0, 1 mM EDTA, 0.3% Tween 20, and 0.3% NP-40). The tubes were then heated to 95°C for 10 min. After cooling, we added 5 μL of Proteinase K (10 mg/mL) to each tube, which were then incubated for at least 1 h at 55°C. After digestion, each tube was tapped to ensure that the tissue was digested. The tubes were then incubated at 95°C for 10 min. Afterward, a 0.5 μL sample was added to a 25 μL PCR reaction and run for 35–40 cycles.

## 3. Results

### 3.1. Clinical presentation

The proband was referred to our hospital at 6 years of age because of global developmental delay. She is the first child of a non-consanguineous Chinese couple. The parents were healthy, without any medical history or inherited disorders. The pregnancy was unremarkable in the first and second trimester. Ultrasound examination in the third trimester, at 29^+4^ gestational weeks (GW) revealed polyhydramnios (amniotic fluid index 255 mm) and a slight widening of the lateral ventricle, to 10 mm. The baby was born at 41 GW via cesarean section, due to cephalopelvic disproportion. At birth, her head circumference (HC) was 36 cm (+1.75 SD), and her birth weight (BW) was 3.6 kg (+0.8 SD). As indicated by meconium-stained amniotic fluid, the baby suffered neonatal pneumonia and jaundice during the neonatal period, which later resolved.

At 4 months old, the baby had severe developmental delay; her intellectual and motor assessments were equivalent to age 0. She could not raise her head or roll over until she was 1 year old. At 1.5 years, she was able to sit independently for less than 1 min. Until she reached 6 years old, she was not able to stand or walk independently (Supplementary Figure S1). Around 2 years old, she started to show visual fixation and tracking, but no response to small objects. She spoke only in single words, had poor social interaction, and had hypertonia in the extremities and brisk deep tendon reflexes. She also showed involuntary, repetitive vertical head movements, and video electroencephalogram monitoring showed no seizures or epileptiform activities. The clinical and genetic features of the patient are summarized in Table 1; Supplementary Figure S1.

**Table 1:** Summary of clinical manifestations and genetic variants.

	P1(CH3101)[2]	P2(CH3102)[2]	P3(CH3105)[2]	P4(CH2401)[2]	P5(CH2402)[2]	P6(CH2701)[2]	P7(M9300017)[3]	P8 (This patient)	Frequency
*CHMP1A* variants	c.28-13G > A(hom)	c.28-13G > A(hom)	c.28-13G > A(hom)	c.88C > T (p.Q30*)(hom)	c.88C > T (p.Q30*)(hom)	c.88C > T (p.Q30*)(hom)	c.28-13G > A(hom)	c.53 T > C (p.Leu18Pro)(het), exon 1 del(het)	
Ethnicity	Peruvian (consanguineous)	Peruvian (consanguineous)	Peruvian (consanguineous)	Puerto Rico (non-consanguineous)	Puerto Rico (non-consanguineous)	Puerto Rico (non-consanguineous)	Persian (consanguineous)	Chinese (non-consanguineous)	
Sex	F	M	M	F	F	F	F	F	
Age of reported	16Y	11Y	7Y	4Y	11 M	7Y	9Y	6Y	
Pregnancy complication									
Full term pregnancy	33 weeks, CS	+	+	36 weeks, CS	+	+	NA	+	5/8
Polyhydramnios	−	−	−	−	−	−	−	**+**	1/8
Bilateral ventricles widened slightly	−	−	−	−	−	−	−	**+**	1/8
Perinatal period									
Fetal decelerations	−	−	−	−	+	−	−	−	1/8
Low Apgar scores	−	−	−	−	−	+	−	−	1/8
Neonatal period									
Neonatal pneumonia and neonatal jaundice	−	−	−	−	−	−	−	**+**	1/8
Ventricular septal defect	+	−	−	−	−	−	−	−	1/8
Feeding difficulties	−	+	−	+	−	+	−	−	3/8
Growth delay	+	+	−	+	+	+	+	+	7/8
Microcephaly (age of discovery)	+(16Y)	+(11Y)	−	+(2Y7M)	+(NB)	+(2Y8M)	+(NA)	**−**	6/8
Dysmorphic features									
Asymmetry of the forehead	−	−	−	−	+	−	−	−	1/8
Hollow temples	−	−	−	−	+	−	−	−	1/8
Upturned nares	−	−	−	−	+	−	−	−	1/8
Tented upper lip	−	−	−	−	+	−	−	−	1/8
Low set and posteriorly rotated ears	−	−	−	−	+	−	−	−	1/8
Hypertrichosis	−	−	−	−	−	+		−	1/8
hirsutism, low hair line	−	−	−	−	−	+	+	−	2/8
Dental crowding	−	−	−	−	−	−	−	**+**	1/8
Visual examination									
No measurable vision	−	−	−	−	−	+	−	−	1/8
Poor visual tracking	−	−	−	+	+	+	+	−	4/8
Myopia	+	+	+	−	−	−	−	−	3/8
Astigmatism	+	+	+	+	−	−	−	−	4/8
Esotropia	−	−	+	+	−	−	−		2/8
Strabismus	+	+	−	−	−	−	+	−	3/8
Hyperopia	−	−	−	+	−	−	−	−	1/8
Pseudoptosis	−	+	−	−	−	−	−	−	1/8
Limitation of extraocular movements	−	+	−	−	−	−	−	−	1/8
Nystagmus	−	−	−	−	−	−	+	−	1/8
Cortical visual impairment	−	−	−	+	−	−	−	−	1/8
Digestive system									
Constipation	−	−	−	+	−	−	−	−	1/8
Gastroesophageal reflux	−	−	−	+	−	−	−	−	1/8
Impaired ability to control swallowing	−	−	−	+	−	−	−	−	1/8
Swallowing incoordination and esophageal dysmotility.	−	−	−	−	−	+	−	−	1/8
Vomiting with eating	−	−	−	−	−	+	−	−	1/8
Osteoarticular deformities									
Arthrogryposis multiplex congenita	−	−	−	−	−	+	−	−	1/8
Joint contractures	−	−	−	−	+	+	+	−	3/8
Claw feet	+	−	−	−	−	−	−	−	1/8
Club feet	−	−	−	−	+	−	−	−	1/8
Equinovarus	+	−	−	−	−	−	−	−	1/8
Talipes valgus	−	+	−	−	−	−	−	+	2/8
Joint stiffness	−	−	−	+	−	−	−	−	1/8
Scoliosis	−	−	−	+	−	−	−	−	1/8
Forearm pronation	−	−	−	−	−	−	−	+	1/8
Carpoptosia	−	−	−	−	−	−	−	+	1/8
Knee recurvatum	−	−	−	−	−	−	−	+	1/8
Hypotonia	+	+	+	−	−	+	−	−	4/8
Hypertonia	−	+	−	+	+	+	−	+	4/8
Hyperreflexia	−	+	−	+	+	−	−	+	3/8
Neurologic injuries									
Delay in motor and speech development	+	+	+	+	+	+	−	+	7/8
Walk independently	8 years of age	7y	−	−	−	−	−	−	5/8
Poor social interaction	+	+	+	+	+	+	−	+	7/8
Involuntary repetitive movements	−	+	+	+	−	−	−	+	4/8
Ataxic gait	+	−	−	−	+	−	−	−	2/8
Seizures	−	−	−	−	−	Episodes?	−	−	1/8
Brain MRI									
Small cerebellum (vermis and hemispheres), Small pons	+	+	+	+	+	+	Dandy-Walker anomaly	+	7/8
Small pons	+	+	+	+	+	+		+	7/8
Thin corpus callosum	+	+	+	+	+	+		+	7/8
Myelin dysplasia	+	−	+	−	−	−		+	3/8
Reduced cerebral white matter	+	+	+	+	+	+		+	7/8
Cerebral cortical malformation	−	−	−	−	−	−		−	0/8

CS, cesarean section; NB, new born; NA, not available. The highlight sections are the additional clinical features in this patient.

**Figure 1:**
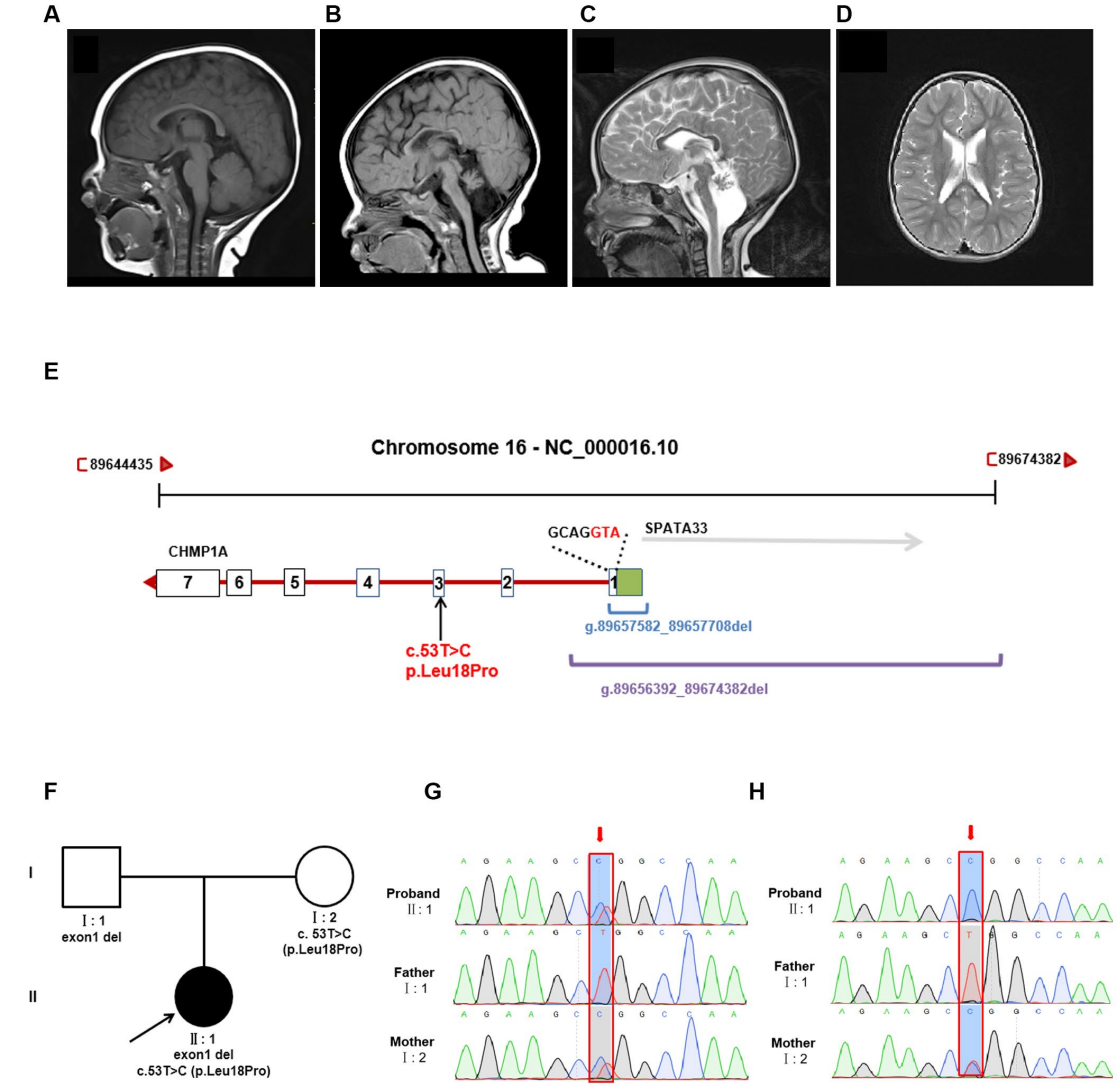
Brain MRI and genetic analysis for two novel *CHMP1A* variants. **(A–C)** T1-weighted sagittal brain MRI images of a normal seven-month-old individual **(A)**, and this patient at seven months **(B)**, and T2-weighted sagittal brain MRI images of this patient at six years of age **(C,D)**. Compared to a control, this patient shows severe hypoplasia of the cerebellum (vermis and hemispheres), small pons, a thin corpus callosum, and mild reduction cortical volume. Hyperintensity in bilateral paraventricular white matter was found, which suggested myelin dysplasia. **(E)** Schematic diagram of two novel *CHMP1A* variants. The purple line represents the entire deletion region (NC_000016.10: g.89656392_89674382del) identified by Whole genome sequencing, which includes the entire exon one of *CHMP1A* (green box represents the untranslated region, the white box represents the coding region, containing seven bases with ATG as the start codon) and a portion of downstream introns, as well as the upstream *SPAT33* gene. The blue line represents the exon one deletion (NC_000016.10:g.89657582_89657708del) identified by whole exome sequencing. The *CHMP1A* gene has seven exons, and another allelic variant c.53 T > C is located in exon three. **(F)** Family pedigree. The proband has two novel variants in *CHMP1A*, one is a missense variant, c.53 T > C(p. Leu18Pro) from the mother, and the other is a deletion of exon one region (NC_000016.10:g.89657582_89657708del) from the father. **(G,H)** Sanger sequencing analysis of variant c.53 T > C(p. Leu18Pro) (red arrow) at DNA level **(F)** and cDNA level **(G)**. From the DNA sequencing, it was evident that the proband sample showed double peaks (T and C) at this site, which indicates a heterozygous variant, whereas in cDNA sequencing, the proband sample showed a single peak of “C,” and the peak of “T” was not shown, which suggests that the cDNA strand of “T”(from the father) had not been transcribed. The sample from the mother showed the same double peaks, “T” and “C,” at this site in both DNA and cDNA sequencing, indicating that both DNA strands were transcribed. As the father has homozygous “T” at this site, only the single peak “T” was apparent in both DNA and cDNA sequencing.

At her 7 months, an MRI of the brain revealed severe hypoplasia of the cerebellum (vermis and hemispheres) and small pons, a thin corpus callosum, and mild reduction cortical volume (Figures 1A,B). A follow-up brain MRI at 6 years of age showed similar cerebellar dysplasia from the initial examination (Figures 1A,C). Hyperintensity in bilateral paraventricular white matter was found, which suggested myelin dysplasia (Figure 1D).

### 3.2. Genetic findings

#### 3.2.1. Whole exome and whole genomics sequencing identification of novel bi-allelic variants in *CHMP1A*

Trio sequencing of the whole exome identified two novel variants in *CHMP1A* (NM_002768.5): one is a novel missense variant, c.53 T > C(p. Leu18Pro)(Hg38 chr16: 89651621) which is inherited from the mother, the other is a deletion of exon one (NC_000016.10:g.89657582_89657708del) which is inherited from the father (Figures 1E,F). These two variants have not been reported in the normal population database (gnomAD, the 1,000 Genomes Project) or local databases. Variant c. 53 T > C is located in exon 3, resulting in a Leu18Pro substitution, and the prediction scores for Leu18Pro mutation were evaluated by SIFT as 0.203 (tolerated), by Mutation Taster as 1.000 (disease-causing), and by PolyPhen-2 as 0.011 (Benign). According to the American College of Medical Genetics and Genomics classification (ACMG), variant c. 53 T > C was classified as “uncertain significance” with one instance of pathogenic moderate evidence (PM2). Exon1 deletion was also categorized as “uncertain significance” with one instance of pathogenic moderate evidence (PM2).

Whole genome sequencing showed that the *CHMP1A* gene exon1 deletion region is NC_000016.10:g.89656392_89674382del, which covers the deletion region identified by whole exome sequencing (NC_000016.10:g.89657582_89657708del) and involves the entire exon one and its upstream region, and entire *SPATA33* gene (Figure 1E). Variant c. 53 T > C(p. Leu18Pro) was also identified by whole genome sequencing (Figure 1E). No other variants were found associated with the proband’s clinical phenotype.

#### 3.2.2. *CHMP1A* exon one region deletion results in impaired expression

qPCR was performed on the cDNA level, suggesting that expression in both the proband and the father was nearly half that of the mother and normal control (Supplementary Figure S2). In order to elucidate the effect on expression, sanger sequencing of exon three was performed in DNA and cDNA, and variant c.53 T > C(p. Leu18Pro) was analyzed. From the DNA sequencing, it was evident that the proband’s sample showed double peaks (T and C) at this site, which indicates a heterozygous variant, whereas in cDNA sequencing, the proband’s sample showed a single peak of “C,” and the peak of “T” was not shown, which suggests that the cDNA strand of “T”(from the father) had not been transcribed. The sample from the mother showed the same double peaks, “T” and “C,” at this site in both DNA and cDNA sequencing, indicating that both DNA strands were transcribed. As the father has homozygous “T” at this site, only the single peak “T” was apparent in both DNA and cDNA sequencing. The Sanger sequencing results are shown in Figures 1G,H.

#### 3.2.3. Zebrafish *chmp1a* gene variation *Leu18Pro* impairs cerebellum development

**Figure 2:**
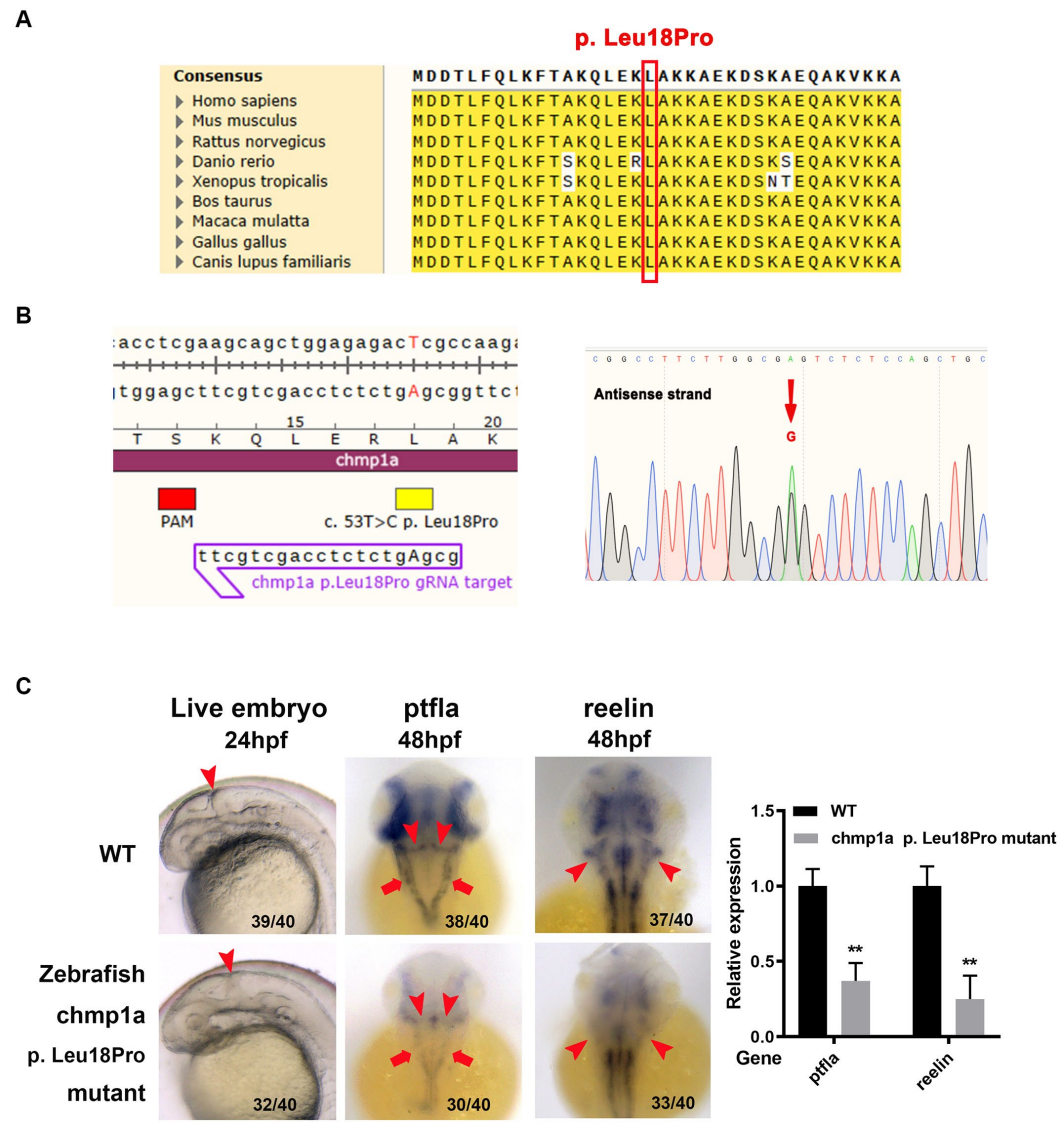
Zebrafish *chmp1a* gene *Leu18Pro* mutation impairs cerebellum development. **(A)** Leu18 in human *CHMP1A* is conserved in species. The site of Leu18 among species is denoted by the red box. **(B)** Construction of *chmp1a* Leu18Pro point mutation zebrafish line by base editor. Schematic diagram (left) and sequencing results (right) of ZSpRY-ABE8e-induced *chmp1a* (Leu18Pro) heterozygous mutation. The protospacer adjacent motif (PAM) sequence is marked by the red box, the mutation information is marked by a yellow box, the detected nucleotide change base A is shown by upper case in the gRNA sequence, the nucleotide substitutions are indicated by a red arrowhead in the sequencing chromatograms. **(C)** Zebrafish *chmp1a* Leu18Pro point mutation impaired cerebellum development. Arrowheads indicate the cerebellum region in live embryo, ptfla, and reelin pictures; arrows in the ptfla picture show the lower rhombic lip (LRL). The percentage and numbers indicated in each picture are the ratio for the number (left in bracket) of affected embryos with a phenotype similar to what is shown in the picture and the total number (right in bracket) of observed embryos. The histogram represents the relative expression of the detected marker gene.

Based on the results of homolog analyses, the amino acid *L18* in human *CHMP1A* has been shown to be conserved in other species, including zebrafish (Figure 2A). To determine whether the *CHMP1A* Leu18Pro variation found in the present study led to defects in cerebellum development, we used the base edition tool adenine base editor ZSpRY-ABE8e (9) to construct a line of zebrafish containing the *chmp1a* gene mutation *Leu18Pro* (Figure 2B).

As detected with the use of a stereomicroscope, the zebrafish embryos with the *Leu18Pro* heterozygous mutation of the *chmp1a* gene showed destroyed cerebellum structure at 24 hpf (hour post fertilization) (Figure 2C). The phenotype resulting from the base edition is comparable to that observed in *chmp1a* knockdown due to morpholino microinjection, as documented by Mochida et al. (2). However, apart from cerebellar abnormalities, we could not observe any other significant abnormal phenotypes in the embryos (Supplementary Figure S3).

Purkinje cells and granule neurons are two major neuronal types in the cerebellum and play important roles in the development and function of the cerebellum. Accordingly, we investigated whether Purkinje cells and granule neurons were impaired in zebrafish embryos with *chmp1a* gene *Leu18Pro* heterozygous mutation. We used the ptf1a gene as a marker of the precursors of Purkinje cells; whole-mount *in situ* hybridization assay showed ptf1a was expressed in the ventricular zone in the wildtype (WT) embryos at 48 hpf. In the embryos with chmp1a gene *Leu18Pro* heterozygous mutation, the expression level of ptf1a was dramatically reduced in the dorsomedial ventricular and ventrolateral zone, including the upper rhombic lip (URL, marked by arrowhead) and lower rhombic lip (LRL, marked by arrow) of the cerebellum (Figure 2C). Furthermore, we found that the expression of differentiated granule cells marker gene reelin was markedly decreased in the cerebellum region (marked by arrowhead) of embryos with the *chmp1a* mutation *Leu18Pro* (Figure 2C). Additionally, we also observed a significant decrease in the expression levels of the nervous stem cell marker, neurog, and the hindbrain neuronal marker, pax2a, at 24 hpf. Simultaneously, the neural plate, marked by the sox3 gene, was disordered in the Leu18Pro heterozygous mutation embryo (Supplementary Figure S3).

## 4. Discussion

Mutations in the human gene *CHMP1A* have been linked to PCH8, through an autosomal recessive inheritance pattern, first reported by Mochida et al. in 2012 and described as a rare genetic disease (2). Since the reporting of these initial findings, Mochida et al. reported similar results in studies of six patients from three families in 2012, there have been no other published reports. There has since been one report of a patient showing severe intellectual disability; however, the study lacked detailed MRI results; thus, information on the state of the cerebellum is lacking (3). If confirmed, that patient would be the seventh known case, and this Chinese patient is the eighth case of PCH8 worldwide, underscoring the rarity of this disorder. Thus, the clinical manifestations and genetic variations for all reported patients are summarized in Table 1.

Clinical manifestations of neurological impairment were evident in all documented cases, including severe growth delay, early motor and speech development delay, no social interaction (in every case, the patient can articulate only single words), abnormal ophthalmologic examination results, hypotonia, hyperreflexia, and skeletal malformations. However, only one of the patients was reported to have had episodes. Microcephaly is also a prominent characteristic; however, only one patient (P5) was found to have an abnormally small head in the neonatal period, other patients were usually in normal range of head circumference (HC) at birth. The age of showing microcephaly is heterogeneous, being present before 2 years of age or after 10 years. With respect to the case reported here, no microcephaly was evident at 6 years of age; microcephaly may be secondary to brain changes as an indicator of PCH8. Abnormal brain MRI findings of these patients are the most characteristic features and the patient described in the present study also showed radiological features similar to those of other reported cases, including severe reduction and asymmetry of the cerebellum (vermis and hemispheres), small pons, a thin corpus callosum, and myelin dysplasia, and no significant change with age (2).

There were some additional features in the clinical phenotype of this patient, which had not been reported before. Dysmorphic features are not typical in all patients, while this child had dental crowding with typical abnormal dentition development and irregular arrangement, which was not described in the reported studies. Most importantly, almost none of the patients showed prenatal characteristics, except for this patient, who had hydramnios, as well as a slight widening of bilateral ventricles in the late-trimester. None of the patients described in published reports had had fetal brain MRI, as this patient did. The earliest brain MRI (in patient 5) was performed at 2 days of age and showed similar typically radiological findings to those found at 10 months of age; thus, cerebellar dysplasia may start in the prenatal stage and does not progress any further after birth. So for a fetus with hydramnios and (or) widening of bilateral ventricles, prenatal brain MRI should be helpful to find the abnormal cerebellar development.

Here, we identified two novel *CHMP1A* variants: the deletion region (NC_000016.10:g.89656392-89674382del) involved the entire exon one of *CHMP1A* and its upstream promoter region and the entire *SPATA33*, which suggests that the variation would affect the transcription of DNA and result in the failure of this DNA strand to be transcribed to mRNA. Sanger sequencing showed the missense variant c.53 T > C(p. Leu18Pro) in exon 3 as heterozygous peaks (T and C) in DNA but a single peak T in cDNA (Figures 1G,H), which indicates that the paternal DNA strand was not transcribed at all, only the maternal DNA strand was transcribed. These findings, combined with the qPCR results, lead us to speculate that the paternal DNA strand with the deletion region probably affects the transcription of this DNA strand, resulting in gene dysfunction. Another missense mutation c.53 T > C(p. Leu18Pro) has been shown to have changed evolutionarily highly conserved amino acid residues in more than one species. To evaluate the functional consequences of this *CHMP1A* variant, the base editing experiment in zebrafish was performed, and a destroyed cerebellum structure was observed in the zebrafish embryos. Further investigations showed the *Chmp1a* expression of Purkinje cells and differentiated granule cells was dramatically reduced in the dorsomedial ventricular, ventrolateral zone, and cerebellum region (Figure 2). In addition, a significant decrease in the expression levels of the nervous stem cell marker, and the neural plate was disordered in the Leu18Pro heterozygous mutation embryo. This result suggests that the mutation impairs the development of the cerebellum in Zebrafish, which is in line with the clinical phenotypes of cerebellar dysplasia in the human subject described here. As for another gene *SPATA33*, which is also included in the deletion region, there are currently no diseases clearly associated with this gene in the OMIM database. According to existing literature, this gene is thought to be related to spermatogenesis (10). However, our patient in this case is female and does not have any relevant clinical symptoms. Therefore, the patient’s phenotype is likely unrelated to the deletion of *SPATA33*.

Although as many as 11 variants of the *CHMP1A* gene have been reported to be associated with disorders from HGMD^2^ (Supplementary Table S1; Supplementary Figure S4) (2–7), only two variants have been confirmed to be related to PCH8: the nonsense variant c.88°C > T and the splicing variant c.28–13 G > A2. Mochida et al. reported that these two variants result in loss-of-function of human *CHMP1A*, causing reduced cerebellar size (pontocerebellar hypoplasia) and reduced cerebral cortical size (microcephaly). Other nine variants reported in *CHMP1A* are associated with neurological and developmental disorders, including three missense variants linked to autism spectrum disorder (4–6), and six missense variants associated with late-onset Parkinson’s disease (7). However, additional studies are needed to verify the effects of these variants. The *CHMP1A* gene has both nuclear and cytoplasmic/vesicular distributions and acts on multiple functional links, which have been assigned two distinct putative functions: membrane trafficking and cell-cycle progression (11, 12). Therefore, the effect and function of this gene on other diseases remain to be clarified.

In conclusion, in this study we identified two novel mutations of *CHMP1A* compound heterozygous mutations of c.53 T > C(p.Leu18Pro) and exon one deletion and have confirmed that these variants are pathogenic, probably causing PCH8 in the patient. In addition, we documented additional features of the PCH8 phenotype, namely prenatal polyhydramnios and lateral ventricle widening, as well as some postnatal phenotypes that had not been reported in previous cases. The results of our study support the association between bi-allelic pathogenic variants in the *CHMP1A* gene and the clinical characteristics of PCH8 and provide further insights into the phenotype and genotype spectrum of *CHMP1A* variants.

## Data availability statement

The original contributions presented in the study are included in the article/Supplementary material, further inquiries can be directed to the corresponding author.

## Ethics statement

The studies involving humans were approved by the Medical Ethics Committee of West China Second University Hospital, Sichuan University. The studies were conducted in accordance with the local legislation and institutional requirements. Written informed consent for participation in this study was provided by the participants’ legal guardians/next of kin. Written informed consent was obtained from the individual(s), and minor(s)’ legal guardian/next of kin, for the publication of any potentially identifiable images or data included in this article.

## Author contributions

XZ, TH, and HS contributed to the study conception and design and wrote the first draft of the manuscript. XZ, TH, HS, BX, HQ, XC, HZ, YL, and ZL performed the material preparation, data collection, and analysis. All authors read and approved the final manuscript.

## Funding

This work was supported by the Sichuan Science and Technology Program (2022NSFSC0658), the National Natural Science Foundation of China (82271692), and the Fundamental Research Funds for the Central Universities (SCU2022F4080).

## Conflict of interest

The authors declare that the research was conducted in the absence of any commercial or financial relationships that could be construed as a potential conflict of interest.

## Publisher’s note

All claims expressed in this article are solely those of the authors and do not necessarily represent those of their affiliated organizations, or those of the publisher, the editors and the reviewers. Any product that may be evaluated in this article, or claim that may be made by its manufacturer, is not guaranteed or endorsed by the publisher.

## Supplementary material

The Supplementary material for this article can be found online at: https://www.frontiersin.org/articles/10.3389/fneur.2023.1228218/full#supplementary-material

## Footnotes

^1^ https://www.genscript.com.cn/

^2^ http://www.hgmd.org
