# Supplementary material for: Novel bi-allelic variants of CHMP1A contribute to pontocerebellar hypoplasia type 8: additional clinical and genetic evidence
Source: Front Neurol. 2023 Sep 18;14:1228218. doi: 10.3389/fneur.2023.1228218 (PMC10544971; doi:10.3389/fneur.2023.1228218)
Supplement: Supplementary file 2 [file Presentation_1.pptx]

## Slide 1
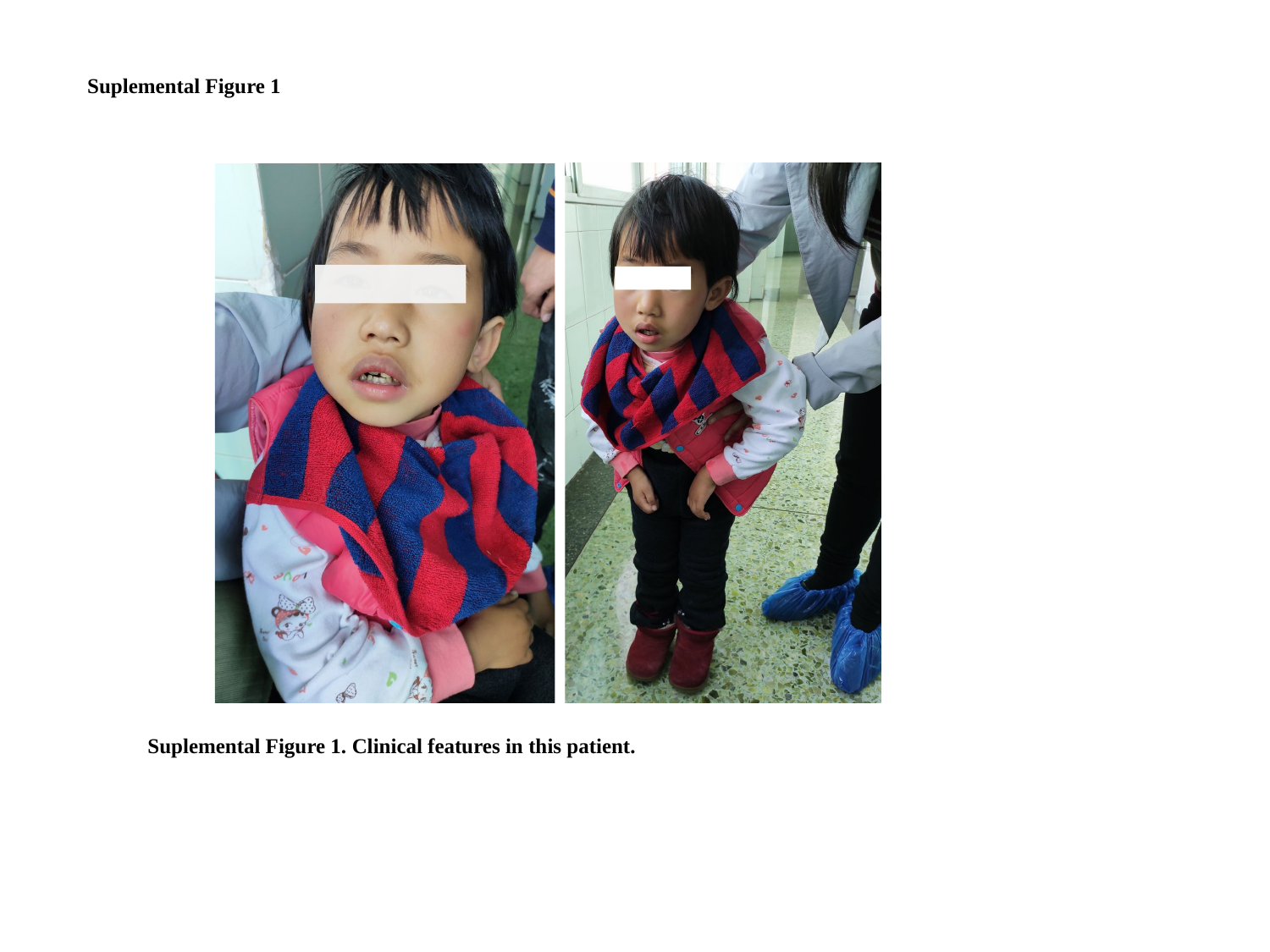

Suplemental Figure 1
Suplemental Figure 1. Clinical features in this patient.

## Slide 2
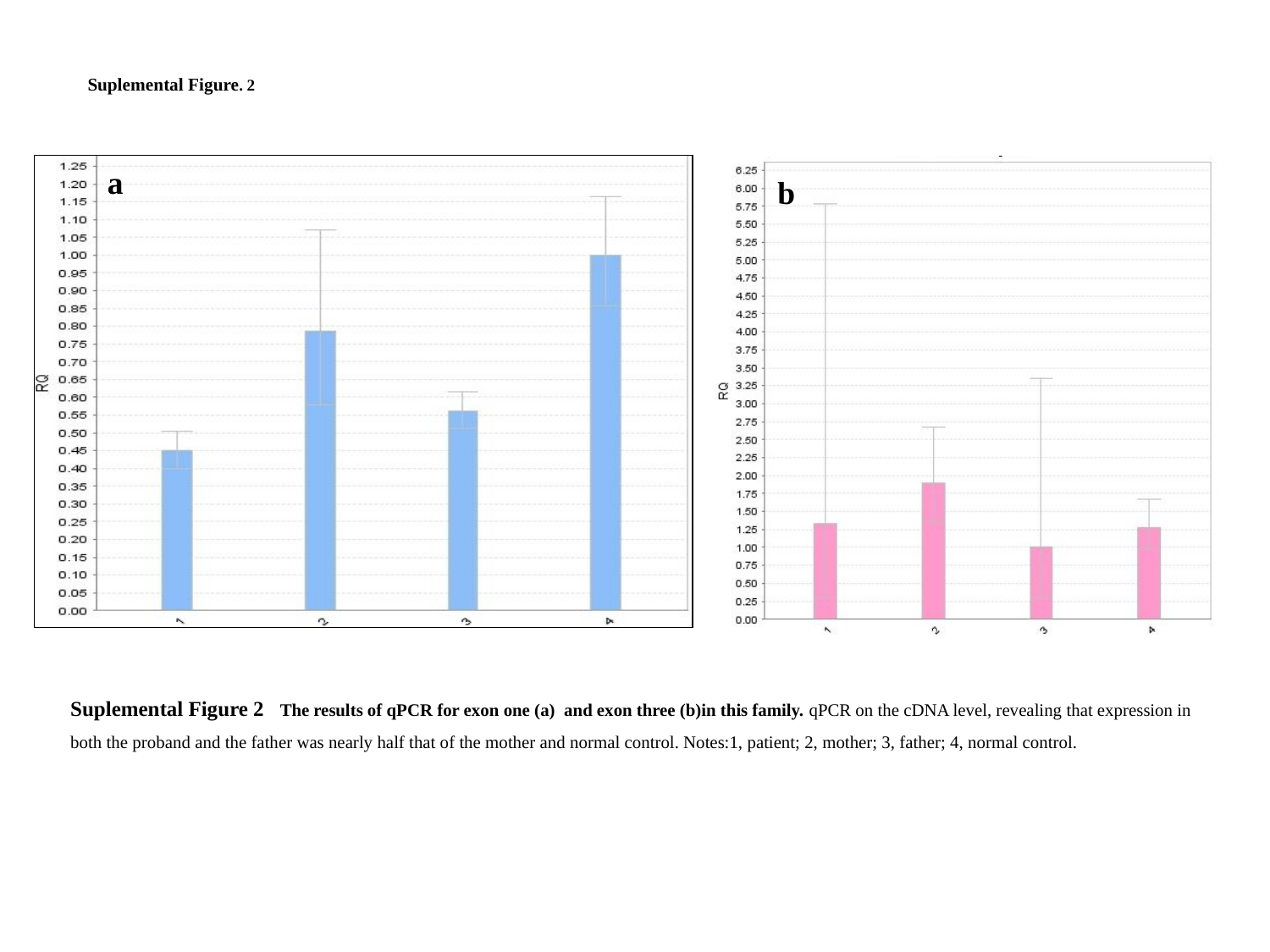

# Suplemental Figure. 2
a
b
Suplemental Figure 2 The results of qPCR for exon one (a) and exon three (b)in this family. qPCR on the cDNA level, revealing that expression in both the proband and the father was nearly half that of the mother and normal control. Notes:1, patient; 2, mother; 3, father; 4, normal control.

## Slide 3
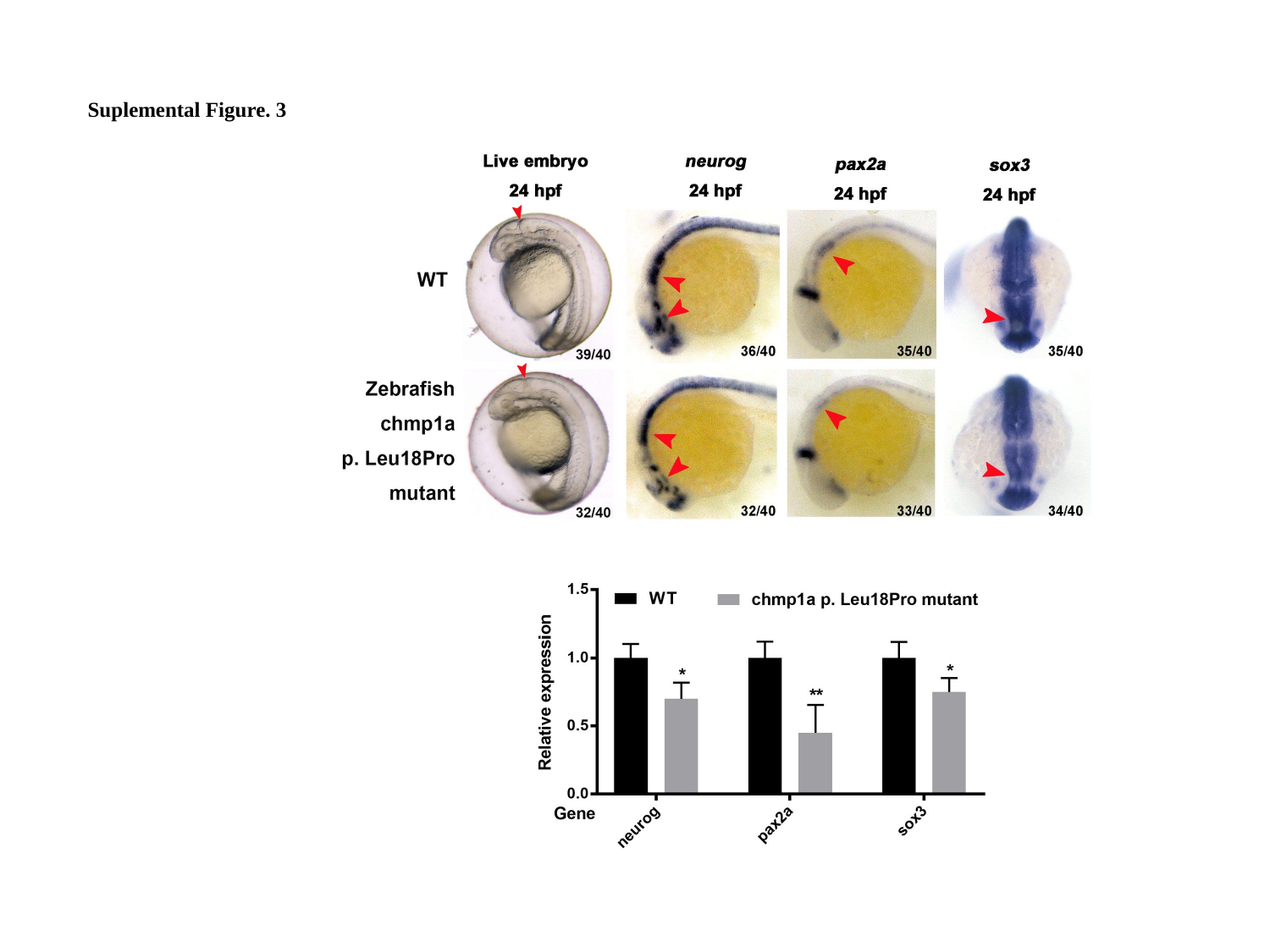

# Suplemental Figure. 3

## Slide 4
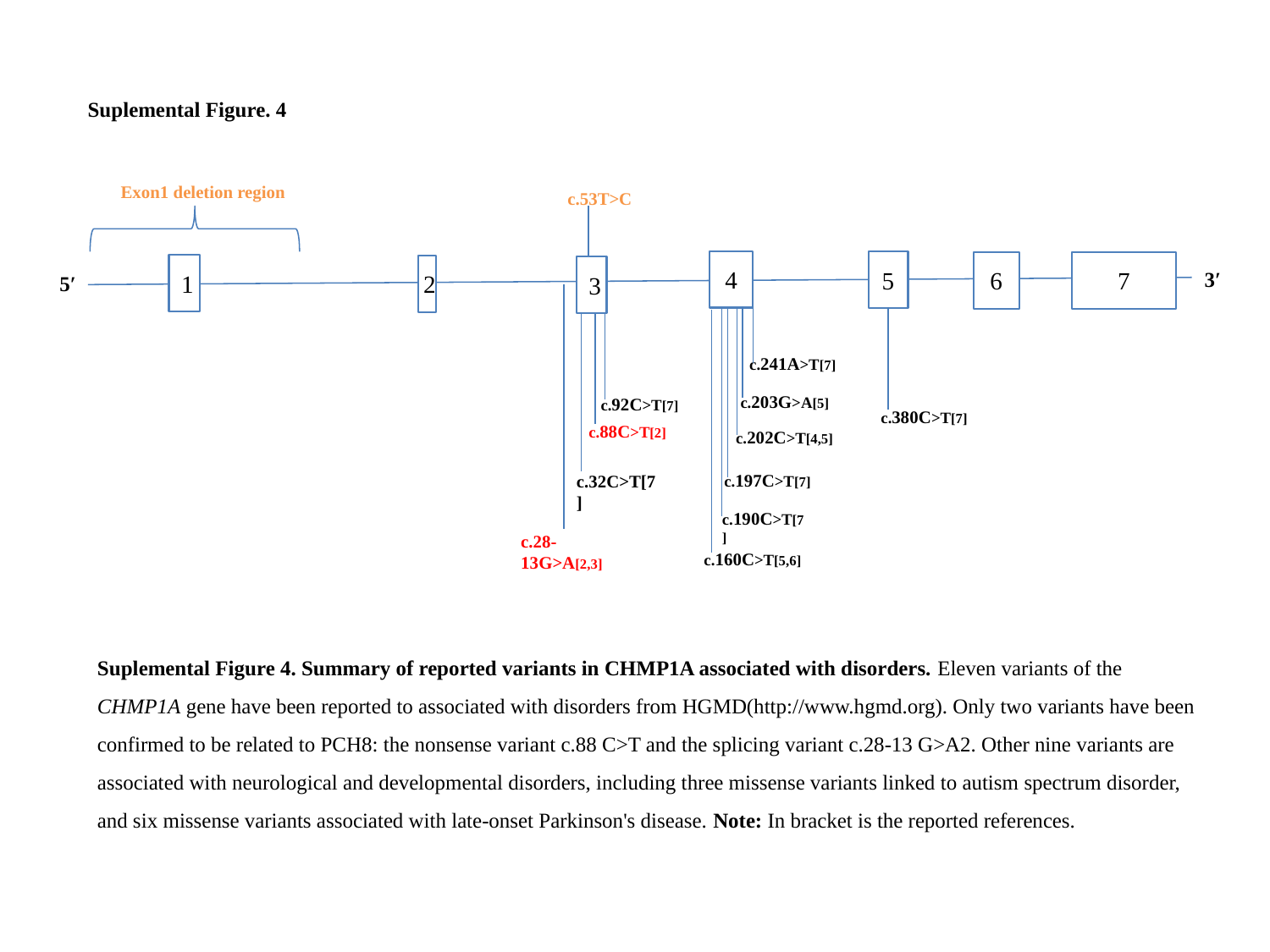

# Suplemental Figure. 4
Exon1 deletion region
c.53T>C
3
c.92C>T[7]
c.88C>T[2]
4
c.241A>T[7]
c.203G>A[5]
c.202C>T[4,5]
c.197C>T[7]
c.190C>T[7]
c.160C>T[5,6]
5
c.380C>T[7]
6
7
1
2
3′
5′
c.28-13G>A[2,3]
c.32C>T[7]
Suplemental Figure 4. Summary of reported variants in CHMP1A associated with disorders. Eleven variants of the CHMP1A gene have been reported to associated with disorders from HGMD(http://www.hgmd.org). Only two variants have been confirmed to be related to PCH8: the nonsense variant c.88 C>T and the splicing variant c.28-13 G>A2. Other nine variants are associated with neurological and developmental disorders, including three missense variants linked to autism spectrum disorder, and six missense variants associated with late-onset Parkinson's disease. Note: In bracket is the reported references.
