# Supplementary material for: Novel bi-allelic variants of CHMP1A contribute to pontocerebellar hypoplasia type 8: additional clinical and genetic evidence
Source: Front Neurol. 2023 Sep 18;14:1228218. doi: 10.3389/fneur.2023.1228218 (PMC10544971; doi:10.3389/fneur.2023.1228218)
Supplement: Supplementary file 3 [file Data_Sheet_1.docx]

**Supplementary materials**

**Supplementary Presentation 1 Methods of Genetic testing**

Trio whole exome sequencing was performed on blood samples from the proband and the parents, using the NanoWES Human Exome V1 (Berry Genomics, Beijing) following the manufacturer's protocol. The DNA libraries after enrichment and purification were sequenced through Illunima NovaSeq6000 platform with 150-bp paired-end reads. The mean depth of coverage of the sequenced sample was 100×. The Burrows-Wheeler Aligner software tool was used for aligning the sequencing reads with hg38/GRCh38, and local alignment and recalibration of base quality of the Burrows-Wheeler aligned reads was performed by the GATK Indel Realigner and the GATK Base Recalibrator, respectively (broadinstitute.org/). Single-nucleotide variants (SNVs) and small insertions or deletions (InDels) were identified by GATK Unified Genotyper (broadinstitute.org/), and functional annotation was performed using ANNOVAR and the Enliven Variants Annotation Interpretation System (Berry genomics). Several public databases were accessed for genome filtering were gnomAD (http://gnomad.broadinstitute.org/), 1000 Genomes Project (1000G) (http://browser.1000genomes.org), and others. The pathogenicity of the detected SNVs was evaluated based on the scientific and medical literature and disease databases, including OMIM (http://www.omim.org), PubMed (https://www.ncbi.nlm.nih.gov/pubmed/), ClinVar (http://www.ncbi.nlm.nih.gov/clinvar), and the Human Gene Mutation Database (HGMD) (http://www.hgmd.org). Variants were classified according to the guidelines of American College of Medical Genetics and Genomics (ACMG). The potential pathogenic variants were validated using Sanger sequencing on an ABI 3500 Genetic Analyzer (Applied Biosystems, Waltham, MA, USA), and the data were evaluated using the Chromas software (2.6.5).

Whole genome sequencing was performed on the sample obtained from proband to confirm the exon1 deletion region and the variant in *CHMP1A* suggested by whole exome sequencing. Using the library construction kit provided by MyGenostics (MyGenostics Inc, Chongqing, China). Quality control of the raw data was performed by removing sequencing junctions, low quality sequences, and short sequences (<80 bp) using cutadaptor software (http://code.google.com/p/cutadapt/). The quality-controlled sequences were then compared to the human genome (UCSC hg19) sequence using BWA software (http://bio-bwa.sourceforge.net/). Picard (http://broadinstitute.github.io/picard/) was used to remove duplicated sequence reads. Single nucleotide polymorphisms (SNPs) loci and insertions-deletions (InDels) were called using HaplotypeCaller of GATK (https://software.broadinstitute.org/gatk/). Filtering of the variants was done using the VariantFiltration function of the GATK software. The filtered data were stored as VCF and annotated using ANNOVA (http://annovar.openbioinformatics.org/en/latest/) software. All candidate variants were filtered against results from public databases, including the 1000 Genome database, ESP6500, dbSNP , EXAC, Inhouse (MyGenostics), HGMD, and other databases. The likelihood that an amino acid transition may affect the function of the protein was estimated by SIFT, PolyPhen-2, MutationTaster, GERP++ and other software. The Copy Number Variants (CNVs) were retrieved using the Control-FREEC software (http://bioinfo.curie.fr/projects/freec/). The CNV data were mapped based on information about their position on the reference genome and compared against searches in databases such as DGV, HDG, and FIG and other databases to annotate the CNV results. CREST (Clipping REvealsSTructure) was used to identify structural variants (SVs) with standard settings.

Additionally, Sanger sequencing of exon 3 at the level of cDNA and DNA was performed using PCR, amplified using 2 × TSINGKE Master Mix (Tsingke, Beijing). The sequences of primers were as follows: cDNA: 5′- AGCTTGGTCGGTTCGATCG -3′ (sense), 5′-CATCCGAAGCCAGTTCACAC-3′ (antisense); DNA: 5′- ACAGAAGACAAACCAGGAG -3′ (sense), 5′- GTTCACAGAGAAGACAGACA -3′ (antisense) (all, Tsingke, Beijing). The PCR products were identified through the use of 2% agarose gel and verified by Sanger sequencing.

**Supplementary Figure legends**

**Supplementary Figure 1. Clinical features in this patient**

This patient was not able to stand or walk independently, and have forearm pronation, carpoptosia, knee recurvatum, and talipes valgus.

**Supplementary Figure 2. The results of qPCR for exon one (a) and exon three (b) of *CHMP1A* in this family**

qPCR on the cDNA level, suggesting that expression in both the proband and the father was nrearly half that of the mother and normal control. Notes:1, patient; 2, mother; 3, father; 4, normal control.

**Supplementary Figure 3. Our established *chmp1a* L18P mutant embryo demonstrated significant dysplasia of the nerve system**

Live embryo is the picture of whole embryo at 24 hpf, arrowheads indicate the cerebellum region, embryo orientation: lateral views with the anterior at the top; nervous system marker genes expression at 24 hpf, arrowheads indicate the different expression sites, embryo orientation: neurog and pax2a, lateral views with the anterior at the left; sox3, head views with the anterior at the bottom; histogram representing the relative expression of the detected marker gene.

**Supplementary Figure 4. Summary of reported variants in *CHMP1A* associated with disorders**

Eleven variants of the *CHMP1A* gene have been reported to associated with disorders from HGMD(http://www.hgmd.org). Only two variants have been confirmed to be related to PCH8: the nonsense variant c.88 C>T and the splicing variant c.28-13 G>A2. Other nine variants are associated with neurological and developmental disorders, including three missense variants linked to autism spectrum disorder, and six missense variants associated with late-onset Parkinson's disease.

Note: In bracket is the reported references.
